# Reducing time-to-unit among patients referred to an outpatient stroke assessment unit with a novel triage process: a prospective cohort study

**DOI:** 10.1186/s12913-018-2952-x

**Published:** 2018-02-27

**Authors:** Maximilian B. Bibok, Kristine Votova, Robert F. Balshaw, Mary L. Lesperance, Nicole S. Croteau, Anurag Trivedi, Jaclyn Morrison, Colin Sedgwick, Andrew M. Penn

**Affiliations:** 1 Department of Research & Capacity Building, Island Health, 1952 Bay Street, Victoria, BC V8R 1J8 Canada; 2 Division of Medical Sciences & Island Medical Program, University of Victoria, PO Box 1700 STN CSC, Victoria, BC V8W 2Y2 Canada; 3 British Columbia Centre for Disease Control, 655 West 12th Avenue, Vancouver, BC V5Z 4R4 Canada; 4 Department of Statistics, University of British Columbia, 3182 Earth Sciences Building, 2207 Main Mall, Vancouver, BC V6T 1Z4 Canada; 5 Department of Mathematics and Statistics, University of Victoria, PO Box 1700 STN CSC, Victoria, BC V8W 2Y2 Canada; 6 Department of Internal Medicine, Section of Neurology, University of Manitoba, 770 Bannatyne Ave, Winnipeg, MB R3E 0W2 Canada; 7 Department of Quality, Safety and Improvement, Island Health, 1952 Bay Street, Victoria, BC V8R 1J8 Canada; 8 Department of Neurosciences, Stroke Rapid Assessment Unit (SRAU), Island Health, 1 Hospital Way, Victoria, BC V8Z 6R5 Canada

**Keywords:** Transient ischemic attack, TIA, Acute cerebrovascular syndrome, ACVS, TIA unit triage, Clinical prediction rule, TIA referral

## Abstract

**Background:**

To evaluate the performance of a novel triage system for Transient Ischemic Attack (TIA) units built upon an existent clinical prediction rule (CPR) to reduce time to unit arrival, relative to the time of symptom onset, for true TIA and minor stroke patients. Differentiating between true and false TIA/minor stroke cases (mimics) is necessary for effective triage as medical intervention for true TIA/minor stroke is time-sensitive and TIA unit spots are a finite resource.

**Methods:**

Prospective cohort study design utilizing patient referral data and TIA unit arrival times from a regional fast-track TIA unit on Vancouver Island, Canada, accepting referrals from emergency departments (ED) and general practice (GP). Historical referral cohort (*N* = 2942) from May 2013–Oct 2014 was triaged using the ABCD2 score; prospective referral cohort (*N* = 2929) from Nov 2014–Apr 2016 was triaged using the novel system. A retrospective survival curve analysis, censored at 28 days to unit arrival, was used to compare days to unit arrival from event date between cohort patients matched by low (0–3), moderate (4–5) and high (6–7) ABCD2 scores.

**Results:**

Survival curve analysis indicated that using the novel triage system, prospectively referred TIA/minor stroke patients with low and moderate ABCD2 scores arrived at the unit 2 and 1 day earlier than matched historical patients, respectively.

**Conclusions:**

The novel triage process is associated with a reduction in time to unit arrival from symptom onset for referred true TIA/minor stroke patients with low and moderate ABCD2 scores.

**Electronic supplementary material:**

The online version of this article (10.1186/s12913-018-2952-x) contains supplementary material, which is available to authorized users.

## Background

The triage of transient ischemic attack (TIA, a cerebrovascular condition often called “mini-strokes”) [1, 2] and minor stroke by outpatient TIA units is a challenging task owing to the urgency with which patients need to be seen, and the limited information often available to guide triage decision making [3]. The focus of triage is to organize and coordinate the risk profiles of multiple patients relative to one another. This coordination is dynamically changing in real-time as new referrals arrive at TIA units and current referrals are seen. Moreover, the risk of recurrent stroke after a TIA/minor stroke is front-loaded [4] with approximately 50% of recurrent strokes that happen within 7 days occurring within the first 24 h of symptom onset [5]. This means that patients’ risk profiles are themselves never static, but constantly changing as a function of time. In regards to the triage of referrals by TIA units, the time elapsed since patients’ index events has to be factored into the triage decision in conjunction with patients’ presenting symptoms. Finally, the dynamic and relative relations between patients’ risk profiles means that all patient referrals, ideally, would need to be evaluated and compared simultaneously when booking unit appointments.

The interplay between symptom presentation and time since symptom onset in defining patients’ potential risk of stroke recurrence after a TIA/minor stroke varies between TIA management guidelines. However, the definition of high risk TIA/minor stroke patients as being those who present with motor or speech focal neurological deficits is consistent across clinical guidelines. This definition is either explicitly stated [3] or implicitly suggested by reference to the ABCD2 score [6]. The ABCD2 score [4, 7] (sometimes referred to as the ABCDD score) is a prognostic marker for risk of recurrent stroke based on five parameters: Age, Blood pressure, Clinical presentation, Duration of symptoms, and Diabetes. Motor and speech deficits comprise the clinical presentation component of the score. The higher the ABCD2 score, the greater the patient’s risk of a subsequent ischemic event.

Slight differences exist among clinical guidelines with respect to the role of time since symptom onset in defining patient risk. Current Canadian guidelines for TIA/minor stroke management recommend that all patients suspected of high risk TIA/minor stroke who present within 48 h of symptom onset should be assessed the same day by stroke experts [3]. The National Institute for Health and Care Excellence (NICE) in the UK has recommended that patients with an ABCD2 score ≥ 4 should be streamlined for early treatment [6]. The American Heart Association recommends that patients with ABCD2 scores ≥4 should be admitted to in-patient stroke units, and those with scores < 3 should be assessed within 72 h of presentation on an outpatient basis [2]. As can be seen, the ABCD2 score plays a prominent role in the application of these recommendations. It is likely for this reason that the ABCD2 score is commonly used to triage patients in specialized TIA units [8].

Unfortunately, such recommendations are of minimal utility for TIA units faced with the realities of patient triage. The issue with triage is not whether an individual patient is in need of treatment (i.e., ABCD2 ≥ 4), but rather which patients are in greater need relative to other patients in a constantly dynamic referral queue. Thus, triage is not about identifying high risk patients, but rather stratifying them. The ABCD2 score is limited in range (0–7) and consequently it is difficult to determine which patients are at greater immediate risk and in need of treatment compared to other patients also deemed to be at risk. Furthermore, a common challenge of TIA/minor stroke triage is the high referral rate of non-cerebrovascular conditions, such as migraine and seizure, which mimic TIA/minor stroke in clinical presentation [9, 10]. Such “mimic” conditions constitute false positive referrals for TIA units, and divert resources from the management of true TIA/minor stroke patients. The ABCD2 score has limited ability to reliably distinguish these mimics from genuine TIA/minor stroke patients in need of urgent intervention [11].

Compounding the challenge of triage in TIA units is the limited clinical information and staff expertise available for decision making. A survey of UK TIA units found that triage is performed by nurses in nearly a third of all units. [8] Implicit to the concept of TIA/minor stroke triage is diagnosis: if a patient experienced a non-cerebrovascular event (i.e., mimic condition), then he or she is not at risk of *recurrent* stroke. Clinic nurses, therefore, in practice, are tacitly charged with diagnosing patients in the course of triage in order to assess risk, even though the task falls well outside their scope of practice. To frame the difficulty of such a task, inter-rater reliability for TIA/minor stroke diagnosis is fairly low even among stroke-trained neurologists with access to complete patient histories [12]. Other than the ABCD2 score, nurses have no existent clinical prediction rules to inform their decisions.

The diagnostic dimension of triage (i.e., strong clinical suspicion) is integral to maximizing the utility of the ABCD2 score in stratifying referred patients. The ABCD2 score is prognostic in nature, which necessarily presupposes a diagnosis of TIA/minor stroke. In the context of TIA/minor stroke triage this entails assuming all referred patients are TIA/minor stroke positive. This assumption, however, is patently false. Referral rates of mimic patients to TIA units are approximately between 40 and 50% [9, 10]. Unless diagnosis is factored into the triage decision, the ABCD2 score will be used outside of the patient population for that which it was intended (i.e., confirmed diagnosed TIA/minor stroke patients). This will effectively decrease the utility of the score as a prognostic indicator of recurrent stroke risk, thereby decreasing the effectiveness of triage within TIA units, and adversely impacting patient outcomes. Conversely, diagnosis by itself is not prognostic: e.g., there may be 100% certainty that a patient had a TIA/minor stroke, but that patient may in fact have a very low prognosis for recurrent stroke risk. Ideally, for triage purposes, the prognostic ABCD2 score should be weighted by the probability of a TIA/minor stroke diagnosis. This would allow the ABCD2 score to retain its prognostic value as an indicator of recurrent stroke risk, while compensating for the presence of mimic patients in the referred population. Such a weighted ABCD2 score could help TIA unit staff to increase the number of referred TIA/minor stroke patients seen in a timely manner, relative to mimic patients, by more effectively stratifying the risk profiles of referred patients.

Toward the goal of assisting clinicians in differentiating TIA/minor stroke from mimic patients (i.e., diagnosing on the basis of predicted probabilities) our research group, Spectrometry in TIA Rapid Assessment (SpecTRA), [13] has developed a clinical classifier (i.e., logistic regression model) based upon presenting clinical symptoms [14]. To remedy the previously discussed triage challenges in our own TIA unit (Stroke Rapid Assessment Unit (SRAU), Victoria, BC, Canada) we developed a novel triage system that combines our previously developed clinical classifier with the ABCD2 score to produce a new weighted triage score. This weighted triage score combines the probability of TIA/minor stroke (diagnosis) with the ABCD2 score (prognosis), while adjusting for time since symptom onset (risk; as the risk of recurrent stroke is greatest immediately post event), to produce a time-dependent, weighted ABCD2 score. Our aims in the current study are to evaluate the effectiveness of our new triage system and score on patient time to TIA unit arrival, relative to patient event date. Specifically, we aim to determine whether referred patients with a final diagnosis of TIA/minor stroke arrived at the unit earlier after their initial event than a comparable cohort of patients assessed at the unit prior to the implementation of the new triage system.

## Methods

To evaluate the effectiveness of the novel triage system, we used a non-concurrent cohort study design comparing a prospectively collected cohort with a historical control cohort. Institutional study approval was granted by the Health Research Ethics board of the Vancouver Island Health Authority.

### Stroke rapid assessment unit and clinical data capture

The SRAU is a fast-track TIA unit located in Victoria, BC on Vancouver Island. It services an outpatient population that is referred to the unit by emergency department (ED) and general practice (GP) physicians. The unit provides care for the majority of the residents on the island (pop. 750,000). Unit staff receive patient referrals and contact patients by telephone to book unit appointments during business daytime hours, Monday through Friday. Referrals that arrive on the weekend via fax are triaged by unit staff on Monday mornings.

Prior to July 2014, the referral fax form used by the unit encapsulated the data elements of the ABCD score [7] (as distinct from the ABCD2 score [4]), in addition to other data elements relevant to triage, such as date and time of event onset and treatments and tests initiated at the time of referral. Clinic staff triaged patients on the basis of their ABCD scores calculated from the referral form fields, with higher scoring patients being prioritized for appointments. A deficiency of this approach was that when multiple patients shared the same ABCD score, staff were unable to determine which of those patients to prioritize.

To remedy this deficiency the unit introduced a new referral fax in July 2014, referred to as the ACVS Assessment Form [15] (ACVS: acute cerebrovascular syndrome [16]). The starting basis for the development of the assessment form was a logistic regression model (i.e., clinical classifier) created by our research group to differentiate mimic and TIA/minor stroke patients on the basis of presenting clinical symptoms [14]. The model was constructed using historical data (*N* = 4187) extracted from the SRAU electronic medical record system (EMR) for patients referred to the unit between January 2008 and December 2011. The model contains 50 main effects and 12 interaction terms, with 31 of the main effects and 8 of the interaction terms being predictive of TIA/minor stroke (i.e., positive coefficients). After derivation of the model, the Assessment Form was constructed as a data capture instrument for the variables needed to use the model. Data elements are represented on the form as checkboxes that are completed by referring physicians. The new Assessment Form also included clinical and operational data elements from the previously described referral form.

After the Assessment Form was developed it was first distributed to emergency departments within the health authority. Later, the form was distributed to GP offices by way of return fax whenever an old referral fax was submitted to the unit.

Concurrent with the deployment of the Assessment Form the SRAU electronic medical record system (EMR) was updated so as to allow for the recording of all data elements on the ACVS Assessment Form. Specifically, each data element on the Assessment Form was matched by a corresponding data element in the EMR. This allowed staff to digitally record verbatim all referral information submitted on the Assessment Form.

### Triage queue process

In November 2014 a novel triage process was implemented in the SRAU to leverage the additional data elements captured by the ACVS Assessment Form, with the goal of standardizing and streamlining the triage process. The new triage process (henceforth, triage queue) consisted of the following information management sub-processes: (a) calculation of the probability of TIA/minor stroke using the clinical classifier, [14] and calculation of the ABCD2 score; (b) calculation of the risk or recurrent stroke using time-dependent models we derived from the literature on ABCD2 scores and stroke risk; [4] (c) calculation of a weighted triage score based upon the previously calculated probability of TIA/minor stroke and risk of recurrent stroke; and (d) rank ordering of patient referrals on the basis of the weighted triage score. Technical details of the new triage process can be found in Additional file 1.

An Excel file was constructed to retrieve read-only data from the triage queue process; all data entry was centralized in the EMR. In this way, unit staff had a real-time snapshot of the state of the unit’s referrals. Due to the real-time, dynamic nature of the triage system, any new referral form added to the EMR would automatically be included and ranked in the Excel file when staff refreshed the spreadsheet. Procedures were also implemented instructing unit staff to prioritize patients at the top of the sorted referral list for appointments. Once unit staff had arranged a patient’s appointment, they could remove the patient from the real-time view by indicating in the EMR that the patient’s appointment had been booked.

### Neurological assessment

Patients who arrive at the unit are examined by the attending neurologist and given a complete neurological examination. Investigations routinely conducted include Holter monitor, electrocardiogram, transesophageal echocardiography, magnetic resonance imaging (MRI), and computed tomography angiography (CTA). Once examinations are completed neurologists render a final diagnosis and chart this in the EMR. Depending upon the investigations conducted, diagnosis may be time-based (clinically supported), tissue-based (radiologically supported), or both.

Patients are given one of three possible diagnoses: (a) Ischemic stroke/TIA (henceforth, TIA/minor stroke), (b) Mimic, and (c) Other. The Other diagnosis encompasses patients who either had non-cerebral ischemic events, such as cranial nerve ischemia, or hemorrhagic stroke. For cases in which an explicit diagnosis has not been recorded in the SRAU EMR, a default null diagnosis of Unknown is assigned to the cases upon data extraction from the EMR. For the purposes of the present analysis, patients with Other and Unknown diagnoses were excluded from analysis, resulting in a final binary diagnosis variable of TIA/minor stroke or mimic (0 = mimic, 1 = TIA/minor stroke).

A proportion of referred patients never attend the unit (i.e., No Shows). Reasons for non-attendance include: (a) patient suffered a recurrent stroke before his or her appointment date, (b) patient refused an appointment, and (c) patient was inappropriately referred and redirected by unit staff to other specialities. As these patients did not see a TIA unit neurologist, their diagnoses are not registered in the SRAU EMR, thus they were excluded from the present analysis.

### Cohort selection

To prospectively examine the performance of the new triage process on unit efficiency two cohorts of patients were constructed: one before implementation of the triage queue in November 2014 and one afterwards.

The first cohort (henceforth, pre-queue) consisted of patients who were referred to the unit between May 2013 and October 2014. The referral date range for this cohort was selected to be as near in time to the implementation of the new triage process as possible, while being long enough to capture normal variations in unit functioning.

The second cohort (henceforth, post-queue) consisted of patients who were referred to the unit between November 2014 and April 2016.

During both time periods the SpecTRA study was actively recruiting stroke patients from the emergency departments on Vancouver Island in a separate study. Patients enrolled in that study received MRI and/or CTA imaging in the ED, as per study protocol, before being referred to the SRAU. This early imaging may have influenced unit staff to preferentially book these patients for appointments, as such imaging would not need to be arranged at the unit. For this reason SpecTRA study patients were excluded from the present analysis.

### Statistical analyzes

The goal of the present analysis aimed to determine the impact of the new triage process on referred-patient time to the SRAU arrival, relative to patient event date. The pre-queue cohort was triaged by unit staff on the basis of ABCD scores, whereas the post-queue cohort was triaged on the basis of the new triage queue process. In order to standardize the comparison of the two triage systems it was decided that each cohort would be stratified in terms of low (0–3), moderate (4–5), and high (6–7) ABCD2 scores and diagnostic category (TIA/minor stroke vs. mimic). ABCD2 scores for the pre-queue cohort were derived by adding patients’ diabetes status to their ABCD score (as record by staff at the time of triage).

A retrospective, descriptive survival analysis was then conducted to compare time to unit arrival from symptom onset within each of the six strata. We selected symptom onset as our index event as symptom onset is a clinically relevant event, unlike date/time of patient referral, as recurrent stroke risk is relative to the initial event and not referral date. Similarly, Canadian Stroke Best Practice Recommendations [3] also index risk of recurrent stroke to the initial event and not presentation date. Our analysis is descriptive as we do not control for patients who failed to arrive at the unit, or who received a diagnosis of Other or Unknown (as previously defined). Data were right censored at 28 days of symptom onset, with a survival event being defined as arrival to the unit. The 28 day interval was selected based on the Canadian Stroke Best Practice Recommendations as the maximum length of time by which suspected low-risk TIA/minor stroke patients ought to have been assessed by stroke specialists. [3]

Differences between survival curves were tested using an equivalent version of the Peto & Peto modification of the Gehan-Wilcoxon test. [17, 18] The Gehan-Wilcoxon chi-squared test was selected rather than the traditional log-rank test as it is known to be more sensitive to occurrences of early survival events. [19] This sensitivity to early events is well suited to our analysis as (a) patient arrivals to the unit are generally front-loaded in time, and (b) our research question anticipates that unit arrivals should occur earlier in time as a consequence of implementing the new triage process in the unit.

Analyses were completed using the tableone (v0.8.1), [20] survival (v2.41.3), [17] and ggplot2 (v2.2.1) [21] libraries in the R statistical language (v3.3.3) [22].

### Variables

The variables of interest in the present study were (a) date/time of symptom onset, (b) date/time of unit arrival, (c) ABCD2 score, and the (d) clinical symptoms and past medical history recorded on the ACVS Assessment Form. [15] These variables were all retrieved from the SRAU EMR. Patients’ time to unit were computed as the number of calendar days between symptom onset and arrival at the unit.

For the post-queue cohort, ABCD2 scores were calculated on the basis of the clinical information recorded on the ACVS Assessment Form. Mean substitution using previously established values for patient age (Mean = 68.277 years) and blood pressure (Mean systolic = 141.606 mmHg; Mean diastolic = 78.042 mmHg) was used in the post-queue cohort dataset to correct for missing values in order to replicate the calculation of ABCD2 scores by the triage queue process as it was implemented [see Additional file 1].

### Sample and missing data

**Fig. 1:**
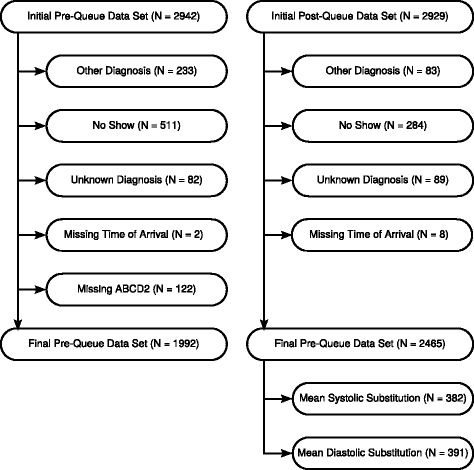
Treatment of missing data for pre- and post-queue cohorts. Pre-queue cohort, May 2013–Oct 2014. Post-queue cohort, Nov 2014–Apr 2016

On initial extraction from the SRAU EMR the pre-queue cohort (May 2013–October 2014) contained 2942 patients and the post-queue cohort (November 2014–April 2016) contained 2929 patients. Both datasets were restricted to patients with a diagnosis of TIA/minor stroke or mimic. Patients missing a date/time for either symptom onset or arrival at the unit were removed from the datasets. Listwise deletion of cases missing ABCD2 scores in the pre-queue dataset was employed. Mean substitution was performed on the post-queue dataset, as previously described. Figure 1 displays the missing data by dataset.

## Results

Table 1 displays the demographic characteristics of the final pre-queue (*N* = 2465) and post-queue (*N* = 1992) datasets after missing data were addressed. Patient characteristics were similar between the two cohorts, though the pre-queue cohort evidenced greater prevalence of TIA/minor stroke, hyperlipidaemia, and hypertension. Although the chi-square of the ABCD2 score indicates that the score differs significantly between the cohorts, the distribution of scores across the range of values (0–7) appears very consistent.

**Table 1:** Demographic characteristics of pre-queue and post-queue cohorts

	Pre-queue cohort (May 2013–Oct 2014)	Post-queue cohort (Nov 2014–Apr 2016)	p*
N	1992	2465	
Patient Age, mean (sd)	70.54 (13.17)	70.31 (13.88)	0.561
Male, N (%)	1013 (50.9)	1218 (49.4)	0.354
Diagnosis of TIA, N (%)	1302 (65.4)	1429 (58.0)	< 0.001
CTA Completed, N (%)	1154 (57.9)	1353 (54.9)	0.045
MRI Completed, N (%)	464 (23.3)	590 (23.9)	0.641
ABCD2, N (%)			
0	18 (0.9)	30 (1.2)	< 0.001
1	102 (5.1)	195 (7.9)	
2	237 (11.9)	471 (19.1)	
3	386 (19.4)	526 (21.3)	
4	531 (26.7)	615 (24.9)	
5	396 (19.9)	377 (15.3)	
6	268 (13.5)	213 (8.6)	
7	54 (2.7)	38 (1.5)	
Systolic BP, mean (sd)	146.12 (34.75)	147.01 (22.95)	0.310
Diastolic BP, mean (sd)	80.21 (17.65)	80.05 (11.34)	0.703
Hypertension, N (%)	1277 (64.1)	1439 (58.4)	< 0.001
Hyperlipidaemia, N (%)	902 (45.3)	974 (39.5)	< 0.001
Atrial Fibrillation, N (%)	249 (12.5)	337 (13.7)	0.269
Diabetes, N (%)	374 (18.8)	428 (17.4)	0.238
Smoking, N (%)	222 (11.1)	296 (12.0)	0.397

*= t and chi-square homogeneity test *p* values

**Fig. 2:**
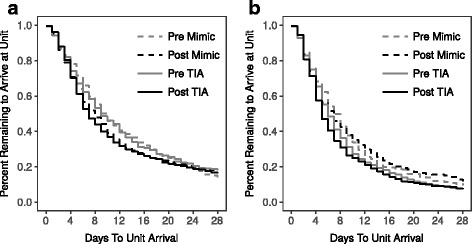
Kaplan–Meier curves of days to unit arrival comparing pre- and post-queue cohorts. Kaplan–Meier curves stratified by diagnosis (TIA/minor stroke vs. mimic), pre- (Pre) and post-queue (Post) cohorts, and (**a**) low risk (0–3) and (**b**) moderate risk (4–5) ABCD2 scores

Table 2 displays the median time to unit arrival from event date among patients seen within 28 days of symptom onset for each ABCD2 risk group, stratified by diagnostic category, along with corresponding Gehan-Wilcoxon chi-squared tests for survival curves. Figure 2a and b displays Kaplan–Meier curves of patient arrival times within 28 days to the SRAU for low and moderate risk ABCD2 scores, respectively.

**Table 2:** Survival curve analysis of days to TIA unit arrival from symptom onset^a^

	Pre-queue cohort		Post-queue cohort			
	N	Median (days)	N	Median (days)	Chi-Squared^b^	*p*
ABCD2—Low (0–3) Mimic	355	10	618	8	3.74	0.053
ABCD2—Low (0–3) TIA	388	9	604	7	4.89	0.027
ABCD2—Moderate (4–5) Mimic	275	7	353	7	0.02	0.887
ABCD2—Moderate (4–5) TIA	652	6	639	5	5.21	0.022
ABCD2—High (6–7) Mimic	60	8	65	5	2.51	0.113
ABCD2—High (6–7) TIA	262	5	186	5	0.58	0.445

^a^Results stratified by ABCD2 score and diagnostic category; data right censored at 28 days

^b^Gehan-Wilcoxon Chi-Squared Test

For the low (0–3) ABCD2 risk group, TIA/minor stroke patients arrived two days earlier after implementation of the new triage process compared to the preceding time period (median 9 vs. 7 days; *p* = 0.027); for mimic patients the difference in unit arrival times was not significant.

For the moderate (4–5) ABCD2 risk group, TIA/minor stroke patients arrived one day earlier after implementation of the new triage process compared to the preceding time period (median 6 vs. 5 days; *p* = 0.022); for mimic patients the difference in unit arrival times was not significant, with median arrival time constant at 7 days across cohorts. This suggests that the distribution of days to unit arrival for TIA/minor stroke patients in these risk groups is different between the pre-queue and post-queue cohorts.

For high (6–7) ABCD2 risk group, mimic patients arrived at the unit 3 days (median) earlier to the unit after the triage process was implemented, although the change in unit arrival time was not statistically significant. Arrival time to unit remained constant for high-risk TIA/minor stroke patients at a median of 5 days.

## Discussion

The goal of the current study was to quantify the impact of a new triage process on time to TIA unit arrival, relative to event date, among ED- and GP- referred patients to one specific TIA unit (the SRAU). Overall, the results of the triage queue process are promising. Implementing the triage queue into the TIA unit was associated with a reduction in patient time to unit arrival by one day for true TIA/minor stroke patients with moderate ABCD2 scores and two days for true TIA/minor stroke patients with low ABCD2 scores. Thus, the patients who need to be seen quickly were, potentially reducing their risk of recurrent stroke by up to 80% [23].

For TIA/minor stroke patients with high ABCD2 scores, median arrival time remained constant at five days across cohorts. From the design of the triage queue, one would expect this group of patients to have arrived at the unit earlier than during the pre-queue period, as well as earlier than the other groups during the post-queue period. We attribute this finding to SRAU staff preferentially booking appointments for patients with high ABCD scores during the pre-queue period. During this period, high risk TIA/minor stroke patients arrived at the unit earlier than all other patients. This suggests that these high risk patients were already effectively triaged during this period, relative to other patients. After implementation of the triage queue, high risk TIA/minor stroke patients still arrived at the unit earlier than other patients, with the exception of mimic patients with high ABCD2 scores (not significant) and TIA/minor stroke patients with moderate ABCD2 scores (statistically significant). This suggests that the relative rank ordering of high risk TIA/minor stroke patients, relative to other patients, remained unchanged after implementation of the triage queue. The significant improvements in unit arrival time, therefore, occurred for TIA/minor stroke patients with low and moderate ABCD2 scores who were not preferentially triaged during the pre-queue period.

A limitation of the present study is that it is unclear how accurately referring physicians completed the ACVS Assessment Form. Each clinical feature on the form represents a variable in the clinical classifier. Several of the variables in the classifier model relate specifically to mimic conditions of TIA/minor stroke. If referring physicians only report symptoms consistent with TIA/minor stroke, then the ability of the classifier to identify mimic patients will be degraded. Future work will seek to determine if interactive, electronic versions of the ACVS Assessment Form will enhance physician reporting of mimic related symptoms. Specifically, we anticipate that if the form can assist physicians in making a differential diagnosis, then mimic related symptoms acquire greater salience with referring physicians.

A second limitation is that it was not possible to isolate the effect of the triage queue process on unit arrival times from all other contributing factors. Such factors include changes in staffing (time and people), unit capacity, neurologist availability, staff behaviour, etc. We also could not control how unit staff responded to various sub-processes of the triage cue. Specifically, the Excel-based triage report centralized and consolidated all the unit’s referrals allowing staff to monitor the unit’s referrals in real-time. Prior to this, unit staff had no means available to obtain a gestalt of all the unit’s referrals, let alone a means to triage referrals in real-time. As such, it is likely that the referral consolidation alone would improve the efficacy and accuracy of patient triage, independent of all the other sub-processes of the triage queue process. For this reason, the results of the present study are only applicable to the triage queue process intervention as a whole, and not any particular sub-process. However, the finding that only moderate risk TIA/minor stroke patients arrived at the unit one day earlier, but not moderate risk mimic patients (median arrival time remained constant), suggests that the triage process has an effect on referral triage that is not reducible to the consolidation of unit referrals.

## Conclusion

The present study is unique in that it focuses specifically on the real-world, clinical utility of the clinical classifier and triage process within a regional health authority that services approximately 750,000 residents. Typically, time to arrival for TIA units can be lengthy due to a high proportion of mimic patient referrals that are given equal priority to true TIA/minor stroke patients for a finite number of unit appointments. Early and rapid medical interventions can improve TIA/minor stroke patient outcomes by reducing the risk of recurrent stroke, [23] which for true TIA/minor stroke patients can be front loaded [4, 5]. Thus, good clinical care management requires a dynamic triage system that shuffles patient mix so that TIA/minor stroke patients in higher risk categories are seen quicker than mimic patients. The present study demonstrates that large, multivariate classifiers for TIA/minor stroke can be successfully used in real-world practice to reduce time to unit arrival for true TIA/minor stroke patients, and thereby potentially improving patient care.

Specifically, we can draw the following conclusions. First, the ACVS Assessment Form [15] which captures the required clinical data for the informational subsystem that informs the triage queue can be completed and adopted by referring physicians in real-world, clinical settings. Second, the information captured on the Assessment Form, when utilized by the triage queue process, was the driver behind enabling true TIA/minor stroke patients to experience earlier unit arrival times, relative to their patient counterparts (mimics) with lower risk profiles. Third, our triage system makes a sharp conceptual distinction between the activities of physician referral and TIA unit triage. The majority of existent TIA/stroke scores (ABCD2, FAST) are physician-centric in that they rely on physicians to calculate the score; hence the need for quick and easy to calculate models. The triage of TIA unit referrals, however, is not the responsibility of referring physicians, but rather TIA units. For this reason, it’s counter-intuitive for TIA units to restrict themselves to simple TIA/stroke scores that are targeted to physicians engaged in point of care decision making. Our ACVS Assessment Form allows referring physicians to provide TIA units with detailed, high quality clinical information, without the burden of calculating any type of TIA/stroke score. In turn, such information when entered into the triage queue processes, results in improved TIA unit triage and better patient outcomes.

## Additional file

Additional file 1: Technical Supplement—Reducing time-to-unit among patients referred to an outpatient stroke assessment unit with a novel triage process: a prospective cohort study. The Technical Supplement details the construction and implementation details for the novel triage system. (PDF 143 kb)
