# Supplementary material for: Reducing time-to-unit among patients referred to an outpatient stroke assessment unit with a novel triage process: a prospective cohort study
Source: BMC Health Serv Res. 2018 Feb 27;18:142. doi: 10.1186/s12913-018-2952-x (PMC6389093; doi:10.1186/s12913-018-2952-x)
Supplement: Supplementary file 1 — Technical Supplement—Reducing time-to-unit among patients referred to an outpatient stroke assessment unit with a novel triage process: a prospective cohort study. The Technical Supplement details the construction and implementation details for the novel triage system. (PDF 143 kb) [file 12913_2018_2952_MOESM1_ESM.pdf]

Technical Supplement—Reducing time-to-unit among patients referred to an outpatient stroke assessment unit with a novel triage process: a prospective cohort study

Maximilian B. Bibok, PhD,[1] Kristine Votova, PhD,[1,2] Robert F. Balshaw, PhD,[3,4] Mary L. Lesperance, PhD,[5] Nicole S. Croteau, MSc,[1,5] Anurag Trivedi, MD,[6] Jaclyn Morrison, MSc,[7] Colin Sedgwick, BSc,[2] Andrew M. Penn, MD[8]

Maximilian.Bibok@viha.ca; Kristine.Votova@viha.ca; Robert.Balshaw@bccdc.ca; mlespera@uvic.ca; Nicole.Croteau@viha.ca; anuragtrivedii@gmail.com; Jaclyn.Morrison@viha.ca; colin.sedgwick@gmail.com; ampenn@mac.com

1. Department of Research & Capacity Building, Island Health, 1952 Bay Street, Victoria, BC, V8R 1J8, Canada

2. Division of Medical Sciences & Island Medical Program, University of Victoria, PO Box 1700 STN CSC, Victoria, BC, V8W 2Y2, Canada

3. British Columbia Centre for Disease Control, 655 West 12th Avenue, Vancouver, BC, V5Z 4R4, Canada

4. Department of Statistics, University of British Columbia, 3182 Earth Sciences Building, 2207 Main Mall, Vancouver, BC, V6T 1Z4, Canada

5. Department of Mathematics and Statistics, University of Victoria, PO Box 1700 STN CSC, Victoria, BC, V8W 2Y2, Canada

6. Department of Internal Medicine, Section of Neurology, University of Manitoba, 770 Bannatyne Ave, Winnipeg, MB, R3E 0W2, Canada

7. Department of Quality, Safety and Improvement, Island Health, 1952 Bay Street, Victoria, BC, V8R 1J8, Canada

8. Department of Neurosciences, Stroke Rapid Assessment Unit (SRAU), Island Health, 1 Hospital Way, Victoria, BC V8Z 6R5, Canada

Corresponding author:
Maximilian Bibok
Department of Research & Capacity Building
Island Health, 1952 Bay Street Victoria, British Columbia, V8R 1J8, Canada
Email: Maximilian.Bibok@viha.ca Phone: 250-882-6216

Technical Supplement—Reducing time-to-unit among patients referred to an outpatient stroke assessment unit with a novel triage process: a prospective cohort study

Overview

In November 2014 a novel triage process was implemented in a regional Stroke Rapid Assessment Unit (SRAU) to leverage the additional data elements captured by the ACVS Assessment Form[1] (ACVS: Acute Cerebrovascular Syndrome[2]), with the goal of standardizing and streamlining the triage process. The new triage process (henceforth, the triage queue) consisted of the following information management sub-processes: (a) calculation of the probability of transient ischemic attack (TIA)/minor stroke using the clinical classifier (i.e., logistic regression model) developed by the SpecTRA project,[3] and calculation of the ABCD2 score[4,5]; (b) calculation of the risk of recurrent stroke using models we derived from the literature on ABCD2 scores and stroke risk;[5] (c) calculation of a weighted triage score based upon the previously calculated probability of TIA/minor stroke and risk of recurrent stroke; and (d) rank ordering of patient referrals on the basis of the weighted triage score. The following document details each of the informational sub-processes involved in the implementation of the triage queue process.

a) Calculation of the TIA/Minor Stroke Probability Score and ABCD2 Score

The TIA/minor stroke probability score is calculated on the basis of a logistic regression model (henceforth, clinical classifier) that was developed as part of the SpecTRA project.[3] The coefficients of the particular model used in the triage process were calculated by fitting the logistic regression model to the combined training and test dataset used in the derivation and evaluation of the model (see Bibok et al. 2016, Table S3).[3]

Both the probability of TIA/minor stroke (henceforth, TIA probability score) and the ABCD2 score are calculated using the clinical information collected from the ACVS Assessment Form[1] which is the referral form for the SRAU, Victoria, BC, Canada. In the event that patient age, sex, or blood pressure (BP) are missing on the Assessment Form, mean value substitution is performed using the values derived from the combined training and test dataset upon which the clinical classifier was fitted[3] (age = 68.277 years, sex = 0.491 [female = 0; male = 1], systolic BP = 141.606 mmHg, diastolic BP = 78.042 mmHg). The ABCD2 score is calculated using the standard formula.[5]

b) Calculation of the Risk or Recurrent Stroke Score

The models used to calculate the risk of recurrent stroke (henceforth, stroke risk decay scores) were derived from the work of Johnston et al. (2007) on the cumulative probability of stroke recurrence stratified by ABCD2 score.[5]

In their work, Johnston and colleagues (p287) present the 2, 7, and 90 days cumulative probabilities of recurrent stroke stratified by low (0–3), moderate (4–5), and high (6–7) ABCD2 scores. From these data it can be observed that the risk of recurrent stroke is front-loaded. The goal of our modelling was to invert these cumulative probabilities, thereby allowing us to model the reduction in risk of recurrent stroke over time; hence, risk decay scores. That is, as the risk of recurrent stroke is front-loaded, the longer an individual goes without experiencing a second event, the less likely it is that a second event will occur.

The generation of risk decay curves was as follows. The data from Johnston et al. (2007) were first graphed in Excel using a trend line to approximate the cumulative probability for each ABCD2 stratification over a 90 day period (i.e., 2, 7, and 90 days) delimited in hours. Several trend line models were visually evaluated to determine which best approximated the data. The

final trend line models selected were an exponential model for the low risk ABCD2 group, and logarithmic models for the medium and high risk groups. These functions were selected as their trend lines (curves) best represented the clinical intuition of patients' risk of recurrent stroke decaying over time in a non-linear fashion.

Using the equations (models) gathered from the trend lines, new graphs were plotted using time in hours as the input variable, beginning with 0 hours and ending at 2160 hours (90 days). The equations were then linearly transformed to achieve the following form-preserving effects: (a) curves were reflected about the x-axis, thereby changing them from curves of cumulative risk to curves of decaying risk; (b) the reflected curves where next vertically shifted such that they were above the x-axis; and finally (c) the y-axis was normalized, such that the high risk group, at time 0 took on a value of 1.0 (i.e., risk was normalized along the y-axis to have a maximum value of 1.0).

The final equations derived for the low, moderate, and high ABCD2 groups, as a function of time (*t*) in hours, are displayed in equations (1), (2), and (3), respectively.

$$(1) \quad y = 0.1408 * \exp(-0.0008t)$$

$$(2) \quad y = 0.6499 - (0.078 * \ln(t))$$

$$(3) \quad y = 0.9999 - (0.131 * \ln(t))$$

In the calculation of the stroke risk decay score for the triage process, time (*t*) is defined as the difference in hours between the date/time of symptom onset reported on the referral form and the current date/time (i.e., whenever the triage system is queried). Our choice of symptom onset as the index event stands in contrast to Johnston et al. (2007) who used the date/time of patient presentation. Our selection was motivated by the following reasons: (a) symptom onset is a clinically relevant event by which to standardize the calculation of the stroke risk decay score

across all patients, unlike date/time of patient presentation; and (b) stroke nurses in the SRAU triage patients on the basis of symptom onset and not date of referral. Figure 1 displays the stroke risk decay scores for the three ABCD2 risk groups, with time represented in days instead of hours for ease of interpretation.

c) Calculation of the Weighted Triage Score and Rank Ordering of Referrals

The weighted triage score is derived by multiplying the TIA probability score and the stroke risk decay score. The rationale for this score was as follows. The ABCD2 score is a prognostic indicator of risk of recurrent stroke following an initial TIA/minor stroke event. As such, the ABCD2 score presupposes the event in question was a TIA/minor stroke, but this cannot be determined until patients have been assessed at the unit. Mimic patients can potentially have high ABCD2 scores due to the risk factor components of the score (e.g., age, blood pressure, diabetes). In contrast, the diagnostic TIA probability score differentiates between TIA/minor stroke and mimic conditions, but itself is not a prognostic indicator of risk. By weighting the stroke risk decay score by the TIA probability score, the risk score of the patient is adjusted by the probability of the patient being TIA/minor stroke. As both scores share the same range (0–1.0) the weighted triage score takes on the same range of values, with 1.0 representing the highest triage urgency and 0 the least. The intention of the weighted triage score was that for matched ABCD2 scores (i.e., risk decay scores), TIA/minor stroke patients would be prioritized over mimic patients for unit treatment.

Once the weighted triage scores are calculated, active referrals are ranked by the triage scores in descending order. Patients at the top of the referral list will have the largest weighted triage scores and will be prioritized for unit appointments.

Implementation of the Novel Triage Process

All information sub-processes described above (a–c) were implemented directly within the SRAU electronic medical record (EMR) database as a single, structured query language (SQL) view. As such, the sub-processes are always executed in real-time at the behest of the staff. When queried the SQL view will: (a) calculate the preceding scores (ABCD2, TIA probability score, risk decay, and weighted triage score), (b) tabulate the scores with the patient demographic (patient name, date of birth, medical record number) and contact information (phone number) entered into the EMR from the referral forms, and (c) sort the tabular result in descending order by the weighted triage scores.

An Excel file was constructed to query the SQL view for read-only, display purposes only; all data entry was centralized in the EMR. In this way, unit staff have a real-time snapshot of the state of the unit's referrals. Due to the real-time dynamic nature of the triage system, any new referral form added to the EMR will automatically be included and ranked in the Excel file when staff refresh the spreadsheet. Procedures were also implemented instructing unit staff to prioritize patients at the top of the sorted referral list for appointments. Once unit staff had arranged a patient's appointment, they can remove the patient from the real-time view by indicating in the EMR that the patient's appointment had been booked.

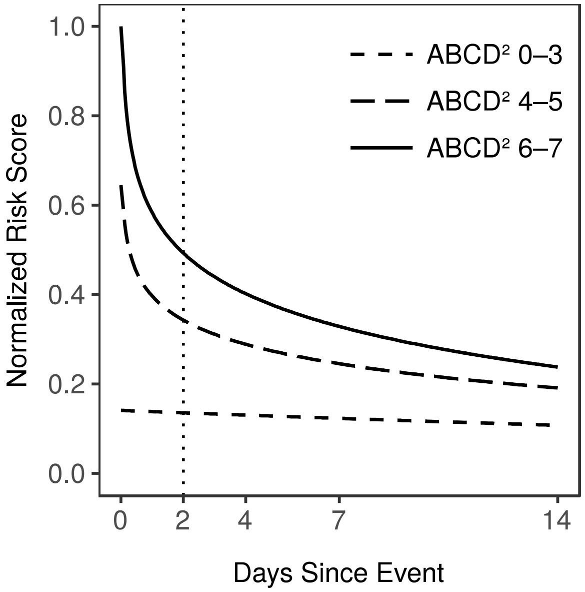

Figure 1. Extrapolated stroke risk decay scores as a function of days since onset. Day 2 indicator included for reference.

References

1. Vancouver Island Health Authority. ACVS assessment form. 2014. doi:10.5281/zenodo.810191.

2. Albers GW. Acute cerebrovascular syndrome: time for new terminology for acute brain ischemia. Nat Clin Pract Cardiovasc. 2006;3:521. doi:10.1038/ncpcardio0679.

3. Bibok MB, Penn AM, Lesperance ML, Votova K, Balshaw R. Development of a multivariate clinical prediction model for the diagnosis of mild stroke/TIA in physician first-contact patient settings. bioRxiv. 2016;. doi:10.1101/089227.

4. Rothwell P, Giles M, Flossmann E, Lovelock C, Redgrave J, Warlow C, et al. A simple score (ABCD) to identify individuals at high early risk of stroke after transient ischaemic attack. Lancet. 2005;366:29–36. doi:10.1016/s0140-6736(05)66702-5.

5. Johnston SC, Rothwell PM, Nguyen-Huynh MN, Giles MF, Elkins JS, Bernstein AL, et al. Validation and refinement of scores to predict very early stroke after transient ischaemic attack. Lancet. 2007;369:283–92. doi:10.1016/S0140-6736(07)60150-0.
